# Supplementary material for: Production, Characterization and Application of a Novel Chitosanase from Marine Bacterium Bacillus paramycoides BP-N07
Source: Foods. 2023 Sep 7;12(18):3350. doi: 10.3390/foods12183350 (PMC10528844; doi:10.3390/foods12183350)
Supplement: Supplementary file 1 [file foods-12-03350-s001.zip › foods-2568297-supplementary.pdf]

Supplementary Table S1：

Table S1. Comparison of properties of purified chitosanases produced by bacteria.

Organism	MW (kDa)	Optimum pH	Optimum temperature (°C)	Composition of products	References
Bacillus lichenformis NBL420	45	8	50	/	[1]
Bacillus mojavensis SY1	31	5.5	55	DP2-4	[2]
Bacillus atrophaeus BSS	27	5	45	DP2-4	[3]
Bacillus glycinifermentans BT2019	32	5	20	DP2-5	[4]
Bacillus amlyoliquefaciens	29	6.5	55	DP2-3	[5]
Bacillus amyloliquefaciens	29	5.6	40	DP2-3	[6]
Bacillus subtilis HD145	31	5.5	50	DP2-4	[7]
Bacillus sp. MD-5					
Bacillus sp. DAU101	35	7.0	40	DP2-3	[8]
Aspergillus fumigatus	27	7.5	50	DP2-4	[9]
CJ22-326	23.5	5.0-6.0	55-65	DP2-6	[10]
Streptomyces albolongus					
ATCC 27414	29.6	8.0	50	DP1-2	[11]
Amycolatopsis orientalis IFO12806	104	5.3	60	/	[12]

1. PYO, H. I.;JANG, H. K.;LEE, S. Y. and CHOI, S. G. Cloning and characterization of a bifunctional cellulase-chitosanase gene from *Bacillus Lichenformis* NBL420. *Journal of Microbiology and Biotechnology* **2003**, *13*, 35-42.

2. Wang, J.;Li, X.;Chen, H.;Lin, B. and Zhao, L. Heterologous expression and characterization of a high-efficiency chitosanase from *Bacillus Mojavensis* SY1 suitable for production of chitosan oligosaccharides. *Frontiers in microbiology* **2021**, *12*, 781138.

3. Cui, D.;Yang, J.;Lu, B. and Shen, H. Efficient preparation of chitooligosaccharide with a potential chitosanase Csn-Sh and its application for fungi disease protection. *Frontiers in microbiology* **2021**, *12*, 682829.

4. Peng, Y.;Wang, Y.;Liu, X.;Zhou, R.;Liao, X.;Min, Y.;Ma, L.;Wang, Y. and Rao, B. Expression and surface display of an acidic cold-active chitosanase in *Pichia Pastoris* using multi-copy expression and high-density cultivation. *Molecules (Basel, Switzerland)* **2022**, *27*, 800.

5. Luo, S.;Qin, Z.;Chen, Q.;Fan, L.;Jiang, L. and Zhao, L. High level production of a *Bacillus Amlyoliquefaciens* chitosanase in *Pichia Pastoris* Suitable for Chitooligosaccharides Preparation. *International journal of biological macromolecules* **2020**, *149*, 1034-1041.

6. Bhuvanachandra, B.;Sivaramakrishna, D.;Alim, S.;Preethiba, G.;Rambabu, S.;Swamy, M. J. and Podile, A. R. New class of chitosanase from *Bacillus Amyloliquefaciens* for the generation of chitooligosaccharides. *Journal of agricultural and food chemistry* **2021**, *69*, 78-87.

7. Kang, L. X.;Chen, X. M.;Fu, L. and Ma, L. X. Recombinant expression of chitosanase from *Bacillus Subtilis* Hd145 in *Pichia Pastoris*. *Carbohydrate research* **2012**, *352*, 37-43.

8. Yang, G.;Sun, H.;Cao, R.;Liu, Q. and Mao, X. Characterization of a novel glycoside hydrolase family 46 chitosanase, Csn-Bac, from *Bacillus* Sp. MD-5. *International journal of biological macromolecules* **2020**, *146*, 518-523.

9. Lee, Y. S.;Yoo, J. S.;Chung, S. Y.;Lee, Y. C.;Cho, Y. S. and Choi, Y. L. Cloning, purification, and characterization of chitosanase from *Bacillus* Sp. DAU101. *Applied microbiology and biotechnology* **2006**, *73*, 113-121.

10. Zhou, J.;Liu, X.;Yuan, F.;Deng, B. and Yu, X. Biocatalysis of heterogenously-expressed chitosanase for the preparation of desirable chitosan oligosaccharides applied against phytopathogeni fungi. *Acs Sustainable Chemistry & Engineering*. **2020**, *8*, 4781–4791.

11. Guo, N.;Sun, J.;Wang, W.;Gao, L.;Liu, J.;Liu, Z.;Xue, C. and Mao, X. Cloning, Expression and Characterization of a Novel Chitosanase from Streptomyces Albolongus Atcc 27414. *Food chemistry* **2019**, *286*, 696-702.

12. Cote, N.; Fleury, A.; Dumont-Blanchette, E.; Fukamizo, T.; Mitsutomi, M.; Brzezinski, R. Two exo-beta-D-glucosaminidases/exochitosanases from actinomycetes define a new subfamily within family 2 of glycoside hydrolases. *Biochem. J.* **2006**, *394*, 675-686.

Supplementary Figure S1：

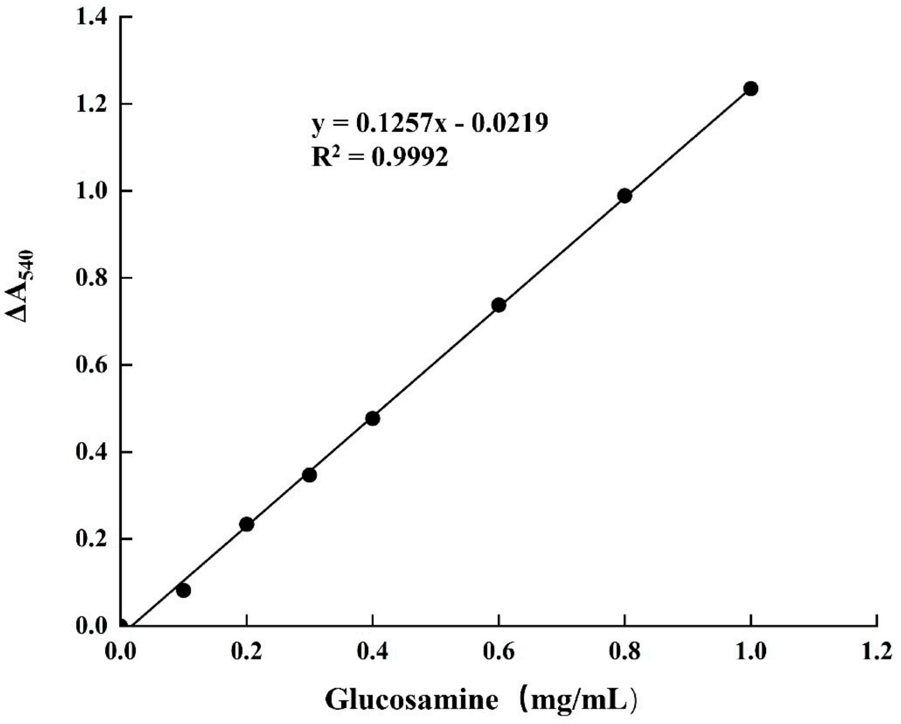

Figure S1. The standard curve of glucosamine (0.1-1.0 mg/mL) for the determination of chitosanase activity.

Supplementary Figure S2:

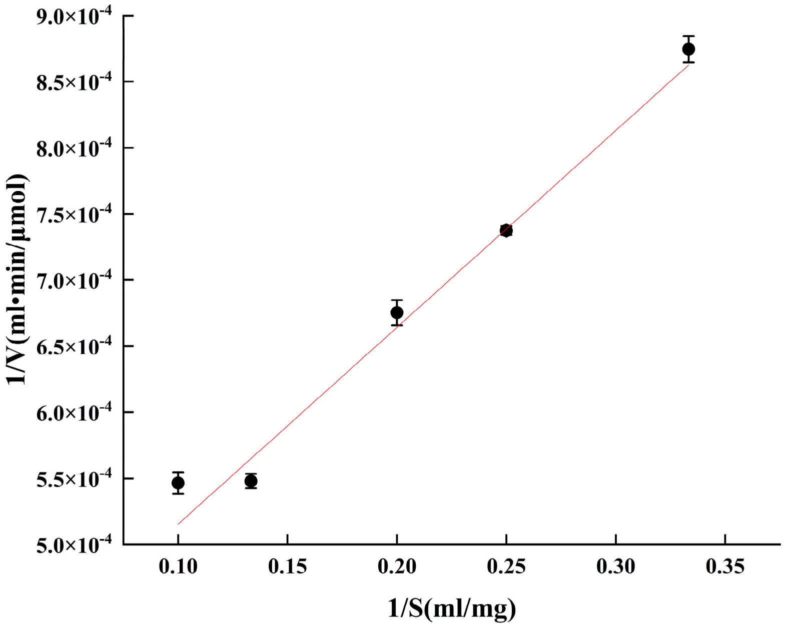

Figure S2. The curve of Lineweaver-Burk. The effects of the chitosan concentration on *Bp*CSN activity. The letters "a,b,c,d" are subsets by Duncan algorithm. The same letters indicate unsignificant difference ($P > 0.05$), the different letters indicate significant difference ($P < 0.05$).

Supplementary Figure S3：

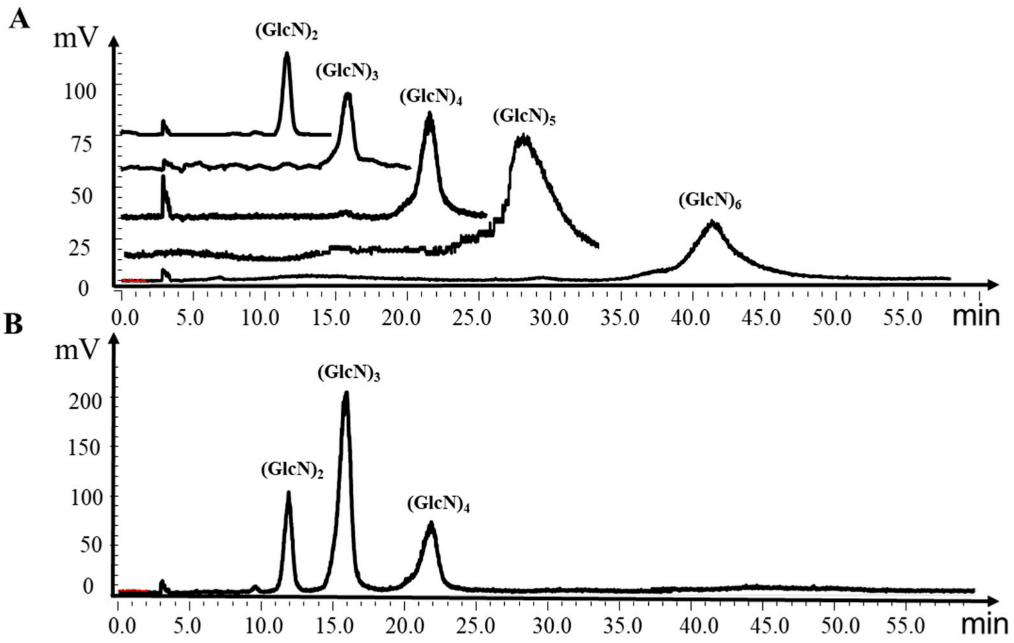

Figure S3. High-performance liquid chromatography (HPLC) analysis of hydrolytic products. A: COSs standard chromatogram diagram. B: HPLC analysis of final products hydrolyzed by *Bp*CSN.

Supplementary Figure S4:

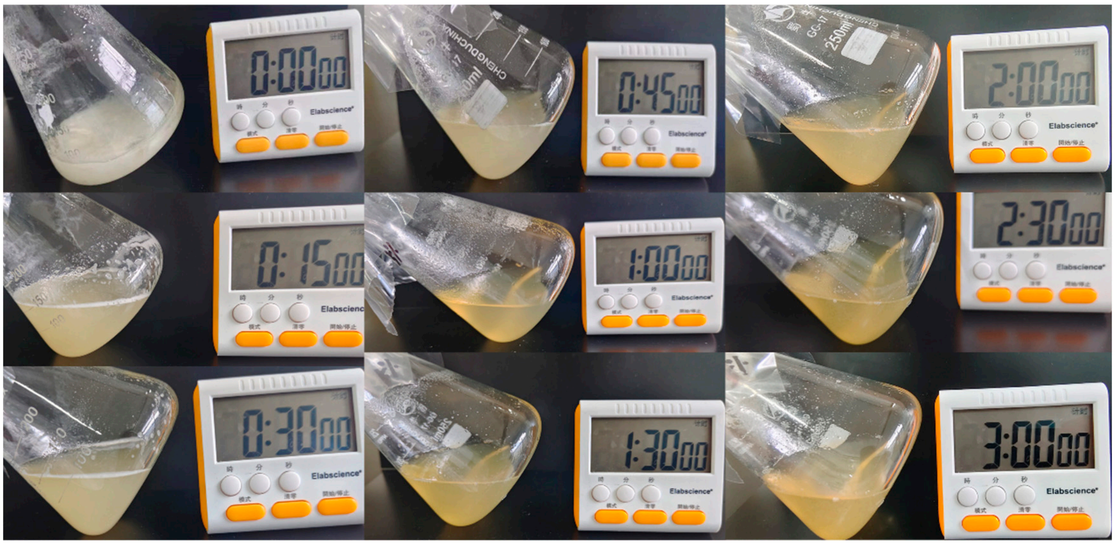

Figure S4. *Bp*CSN was used for a preparation of oligosaccharides.

Supplementary informations:

The 16S rDNA sequences of *Bacillus paramycoides* BP-N07.

>OQ930621.1

CATTGGGGCAGCATAATGCAGTCGAGCGAATGGATTAAGAGCTTGCTCTTATGAAGTTA
GCGGCGGACGGGTGAGTAACACGTGGGTAACCTGCCCATAAGACTGGGATAACTCCG
GGAAACCGGGGCTAATACCGGATAACATTTTGAACCGCATGGTTCGAAATTGAAAGGC
GGCTTCGGCTGTCACTTATGGATGGACCCGCGTCGCATTAGCTAGTTGGTGAGGTAACG
GCTCACCAAGGCAACGATGCGTAGCCGACCTGAGAGGGTGATCGGCCACACTGGGAC
TGAGACACGGCCCAGACTCCTACGGGAGGCAGCAGTAGGGAATCTTCCGCAATGGAC
GAAAGTCTGACGGAGCAACGCCGCGTGAGTGATGAAGGCTTTCGGGTCGTAAAACTC
TGTTGTTAGGGAAGAACAAGTGCTAGTTGAATAAGCTGGCACCTTGACGGTACCTAAC
CAGAAAGCCACGGCTAACTACGTGCCAGCAGCCGCGGTAATACGTAGGTGGCAAGCG
TTATCCGGAATTATTGGGCGTAAAGCGCGCGCAGGTGGTTTCTTAAGTCTGATGTGAAA
GCCCACGGCTCAACCGTGGAGGGTCATTGGAAACTGGGAGACTTGAGTGCAGAAGAG
GAAAGTGGAATTCCATGTGTAGCGGTGAAATGCGTAGAGATATGGAGGAACACCAGTG
GCGAAGGCGACTTTCTGGTCTGTAACTGACACTGAGGCGCGAAAGCGTGGGGAGCAA
ACAGGATTAGATACCCTGGTAGTCCACGCCGTAAACGATGAGTGCTAAGTGTTAGAGG
GTTTCCGCCCTTTAGTGCTGAAGTTAACGCATTAAGCACTCCGCCTGGGGAGTACGGC
CGCAAGGCTGAAACTCAAAGGAATTGACGGGGGCCCGCACAAGCGGTGGAGCATGTG
GTTTAATTCGAAGCAACGCGAAGAACCTTACCAGGTCTTGACATCCTCTGACAACCCT
AGAGATAGGGCTTCTCCTTCGGGAGCAGAGTGACAGGTGGTGCATGGTTGTCGTCAGC
TCGTGTCGTGAGATGTTGGGTTAAGTCCCGCAACGAGCGCAACCCTTGATCTTAGTTG
CCATCATTAAGTTGGGCACTCTAAGGTGACTGCCGGTGACAAACCGGAGGAAGGTGG
GGATGACGTCAAATCATCATGCCCCTTATGACCTGGGCTACACACGTGCTACAATGGAC
GGTACAAAGAGCTGCAAGACCGCGAGGTGGAGCTAATCTCATAAAACCGTTCTCAGTT
CGGATTGTAGGCTGCAACTCGCCTACATGAAGCTGGAATCGCTAGTAATCGCGGATCAG
CATGCCGCGGTGAATACGTTCCCGGGCCTTGTACACACCGCCCGTCACACCACGAGAG
TTTGTAACACCCGAAGTCGGTGGGGTAACCTTTTGGAACCCGCCGGCTAAGGGGGGA
ACGAAGAATGGGGTGGAATCTACAAGGGGTGTCCGCATAAACCC
